# Supplementary figures and images for: The modulatory effects of gut microbes and metabolites on blood–brain barrier integrity and brain function in sepsis-associated encephalopathy
Source: PeerJ. 2023 Mar 28;11:e15122. doi: 10.7717/peerj.15122 (PMC10064995; doi:10.7717/peerj.15122)

A

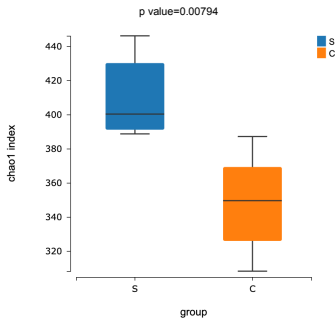

B

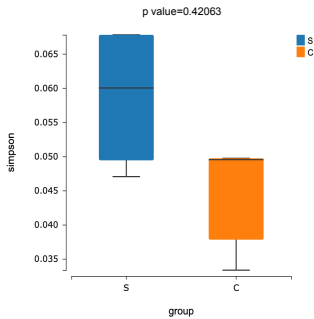

C

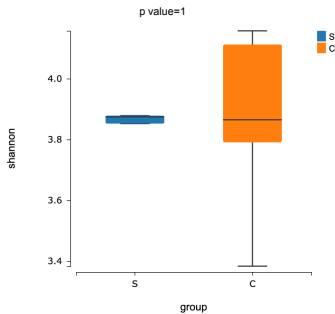

D

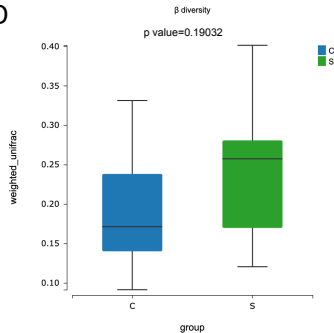

Supplement: Supplemental Information 1 — (A) The Chao1 indexed of gut microbiota was obviously lower in CLP group than that in Sham group (P = 0.00794). (B–C) There is no significant difference between two groups in Simpson (B) and Shannon index (C). (D) There is no significant difference between two groups in β diversity. [file peerj-11-15122-s001.pdf]

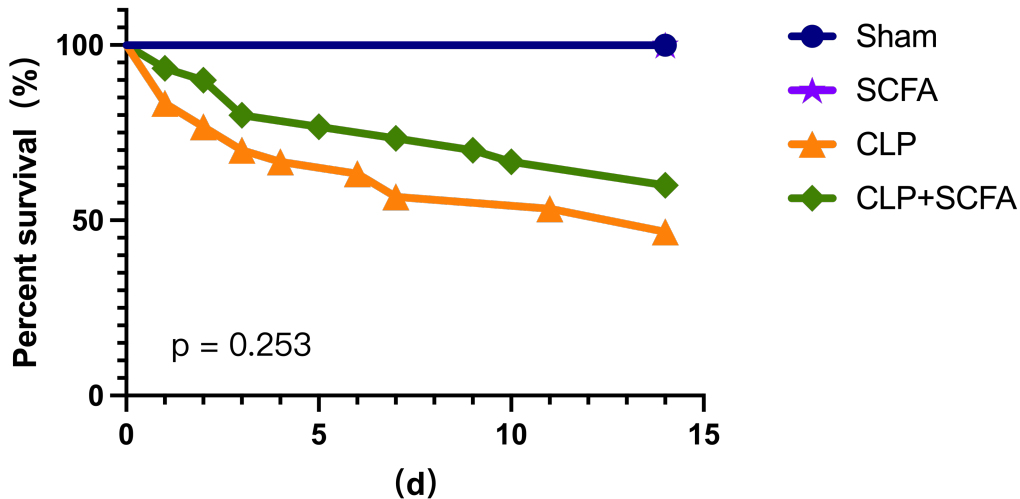

Supplement: Supplemental Information 3 — Values are expressed as survival percentage. (Kaplan Meier method and log-rank test) [file peerj-11-15122-s003.pdf]

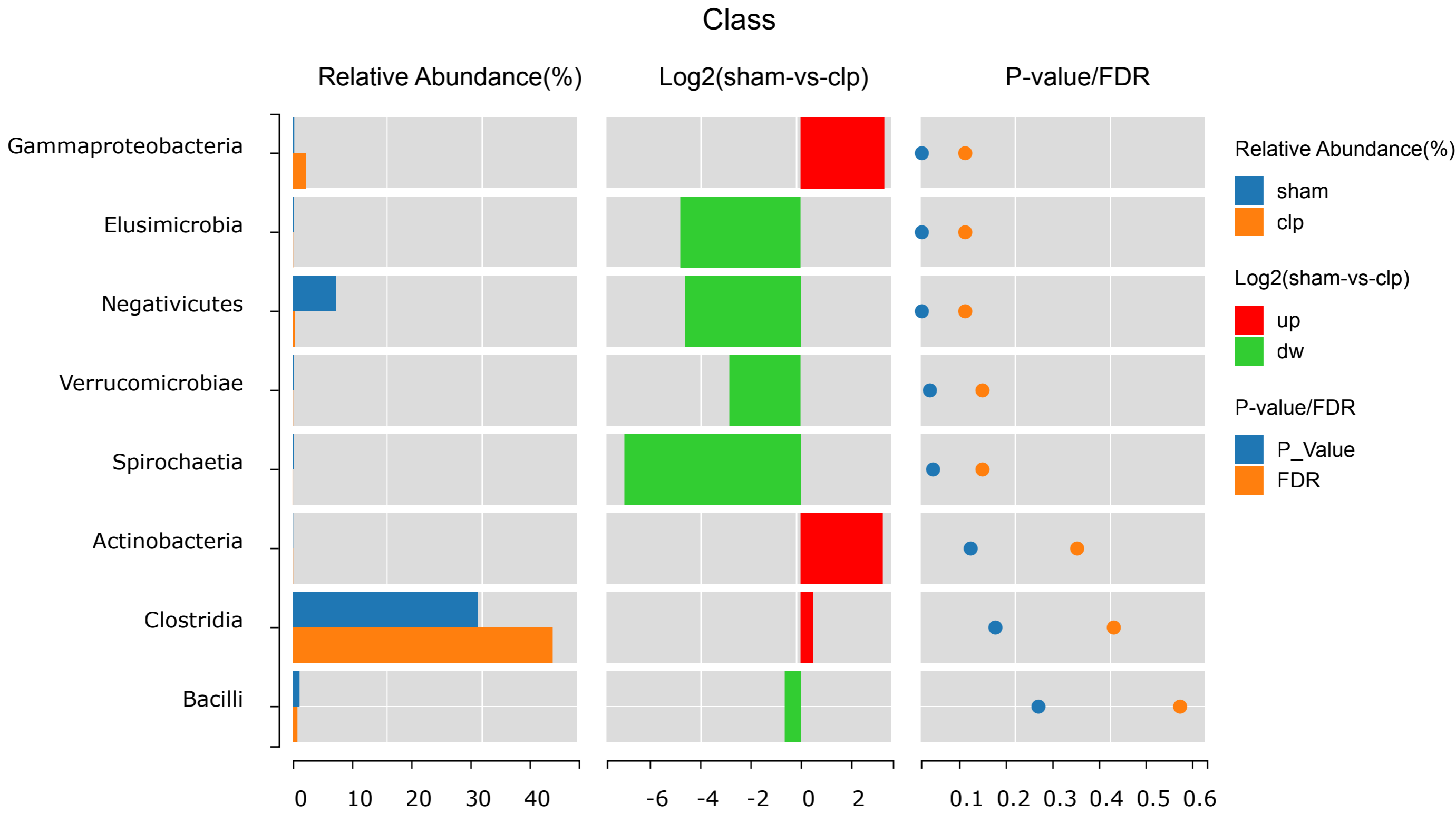

Supplement: Supplemental Information 4 [file peerj-11-15122-s004.zip › Samples without C1/Class.pdf]

# Beta-diversity

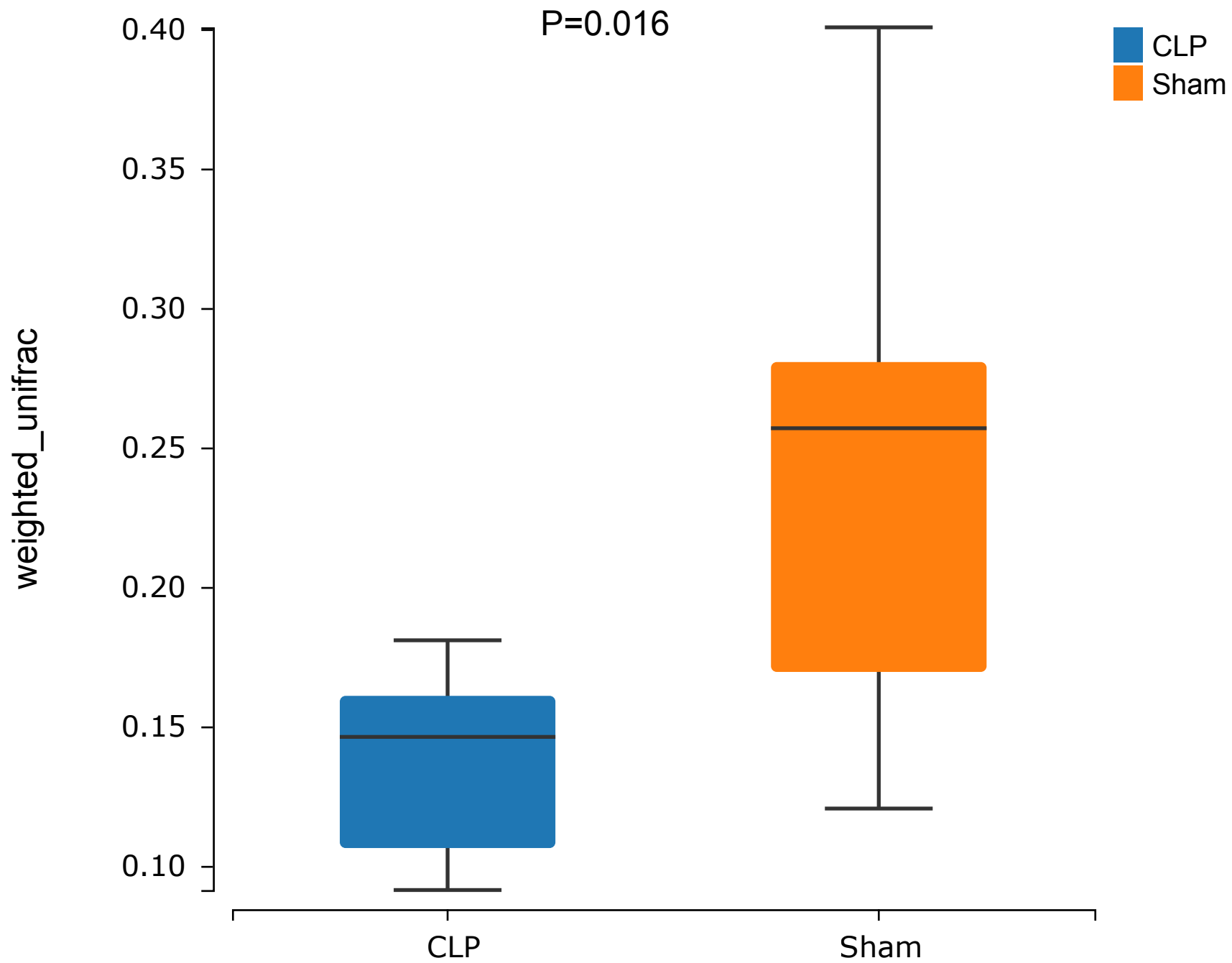

Supplement: Supplemental Information 4 [file peerj-11-15122-s004.zip › Samples without C1/beta-diversity.pdf]

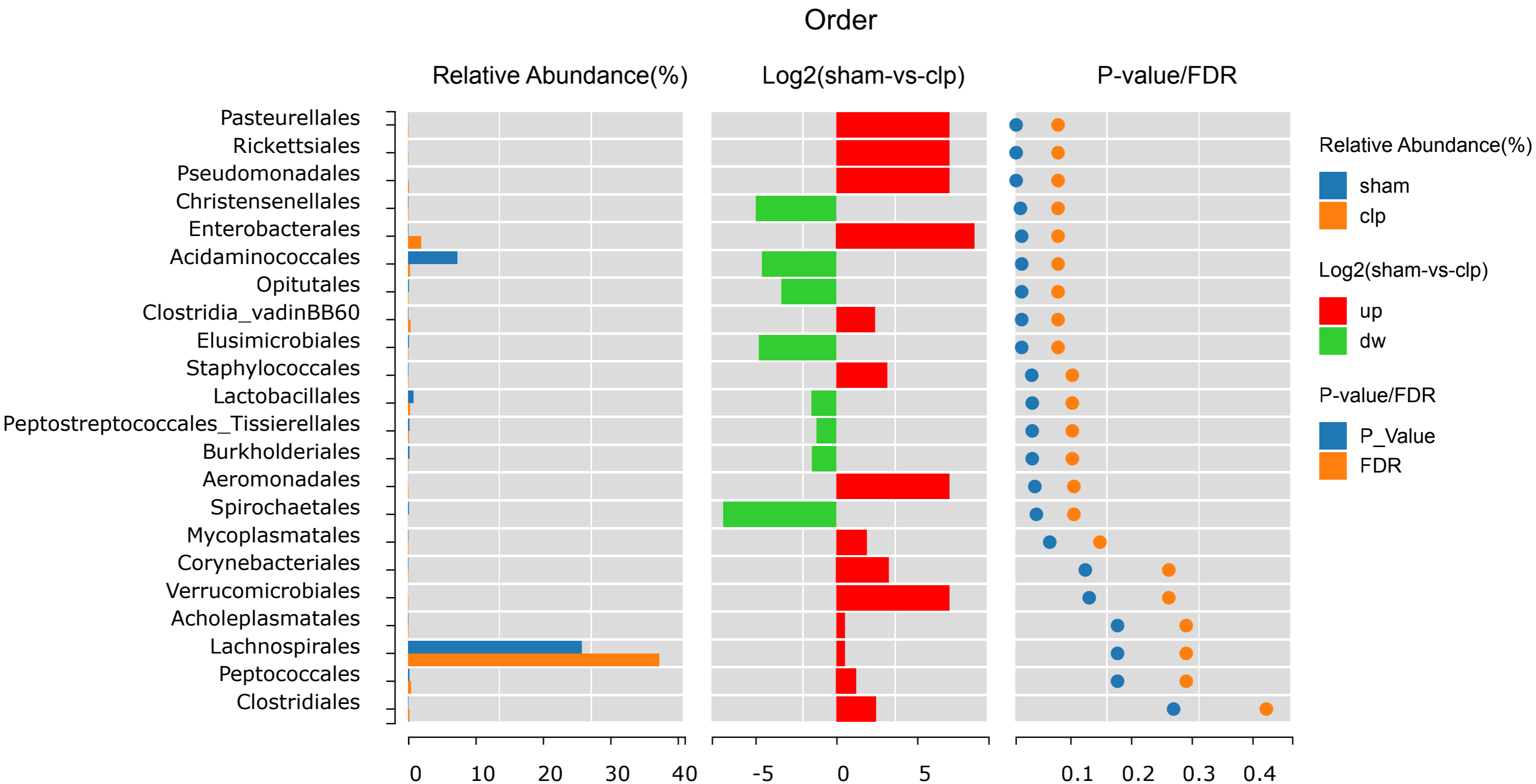

Supplement: Supplemental Information 4 [file peerj-11-15122-s004.zip › Samples without C1/Order.pdf]

P=0.56

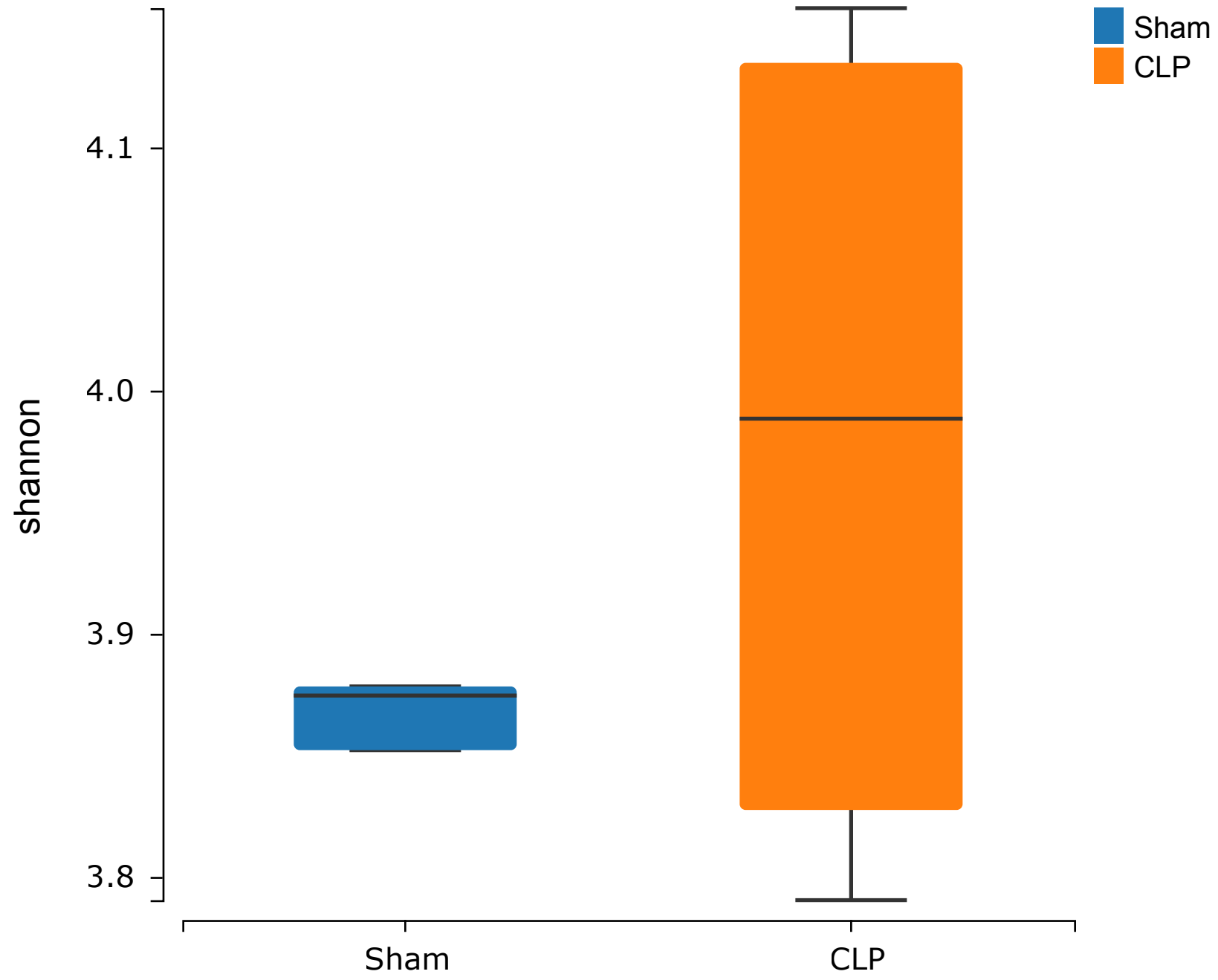

Supplement: Supplemental Information 4 [file peerj-11-15122-s004.zip › Samples without C1/Shannon.pdf]

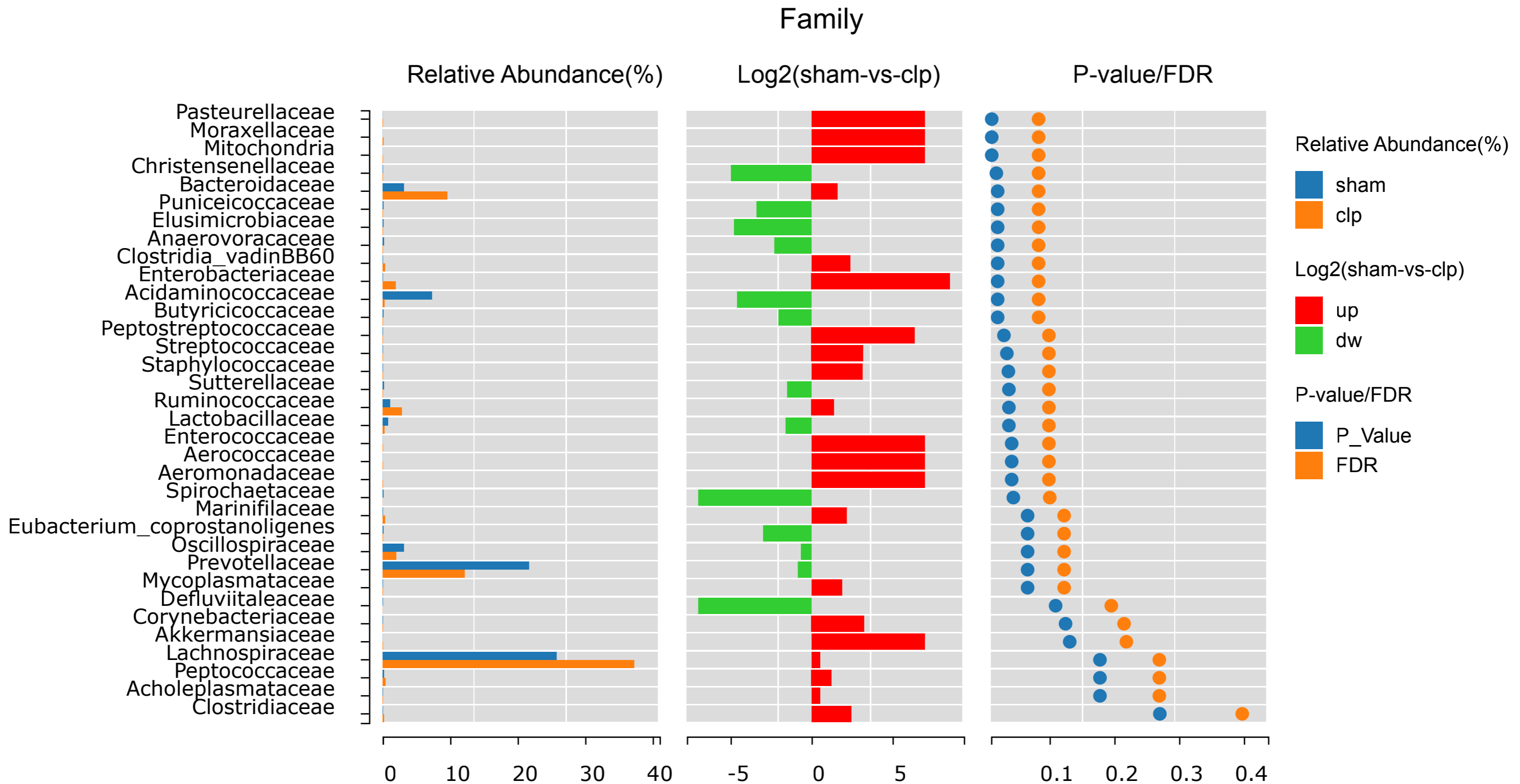

Supplement: Supplemental Information 4 [file peerj-11-15122-s004.zip › Samples without C1/Family.pdf]

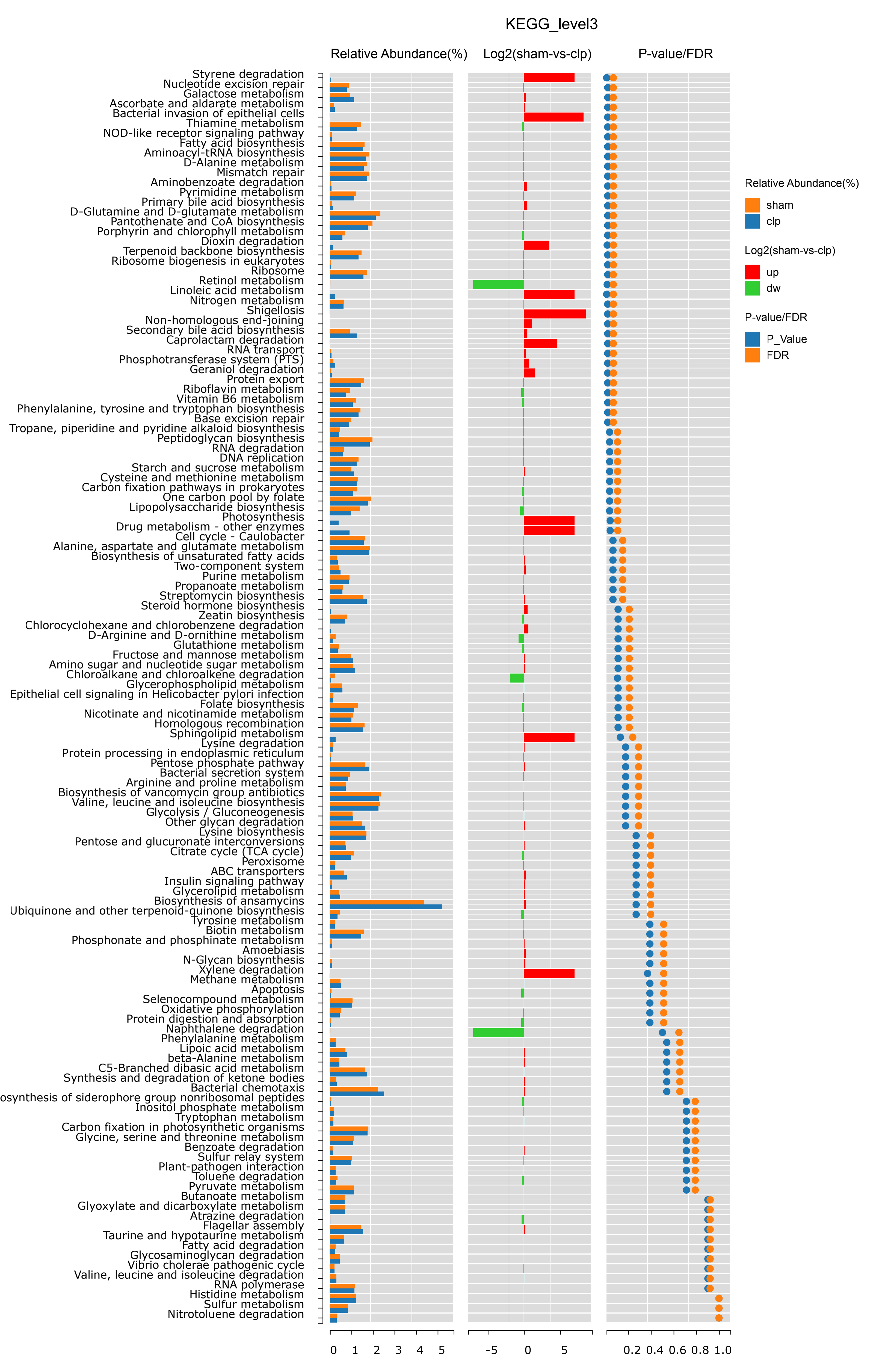

Supplement: Supplemental Information 4 [file peerj-11-15122-s004.zip › Samples without C1/KEGG-level3.pdf]

KEGG\_level2

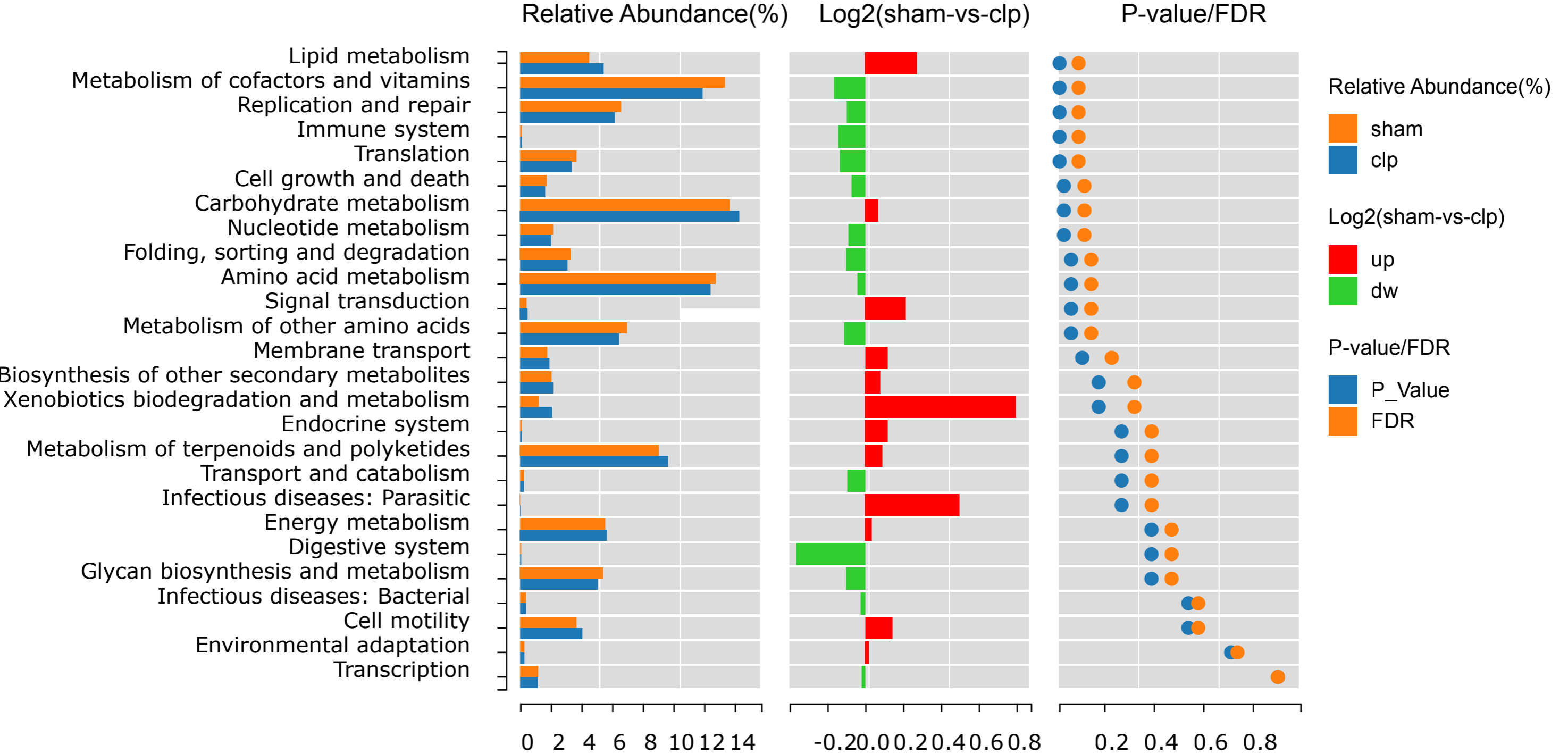

Supplement: Supplemental Information 4 [file peerj-11-15122-s004.zip › Samples without C1/KEGG-level2.pdf]

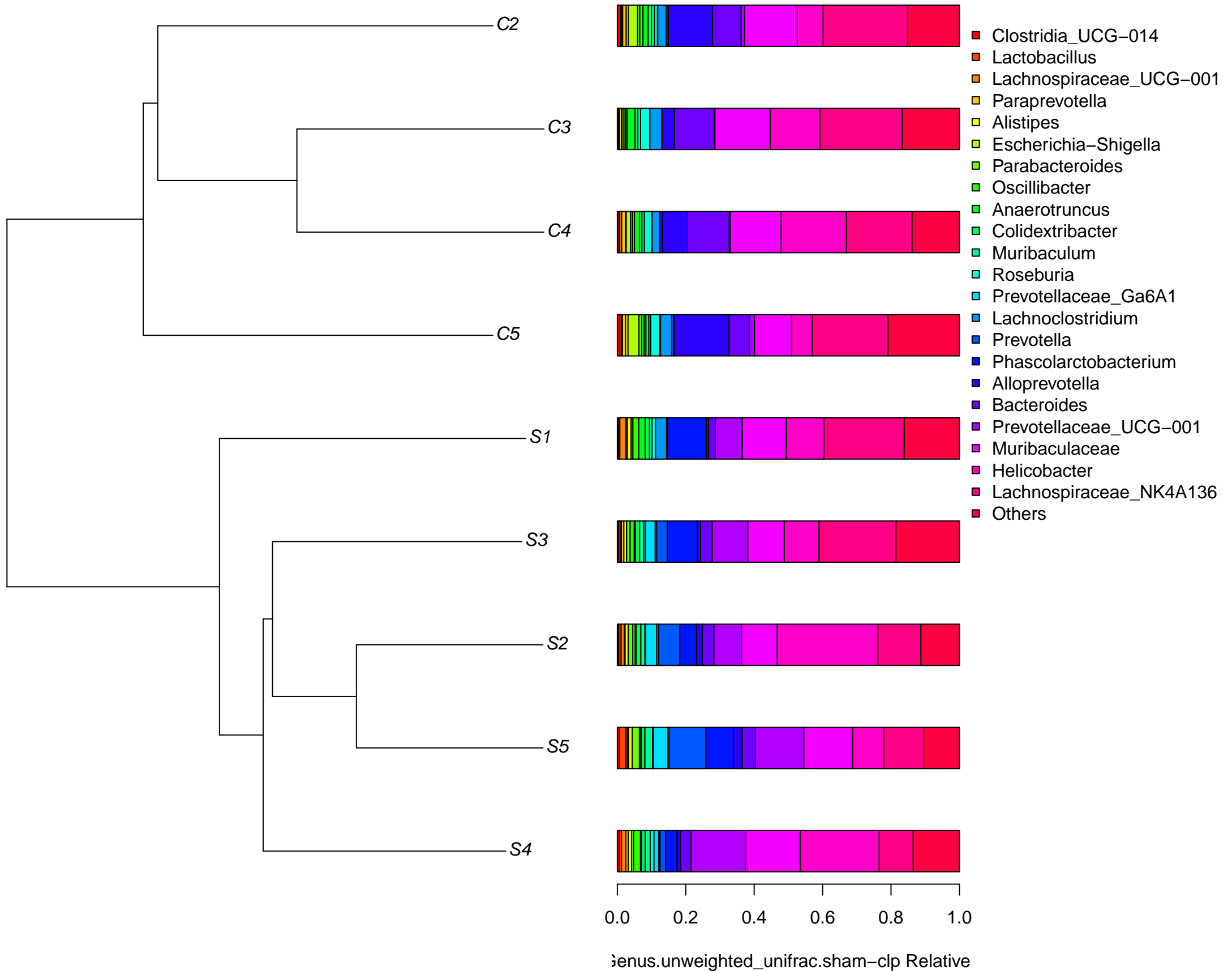

Supplement: Supplemental Information 4 [file peerj-11-15122-s004.zip › Samples without C1/Genus.unweighted_unifrac.sham-clp_taxonomy_tree.pdf]

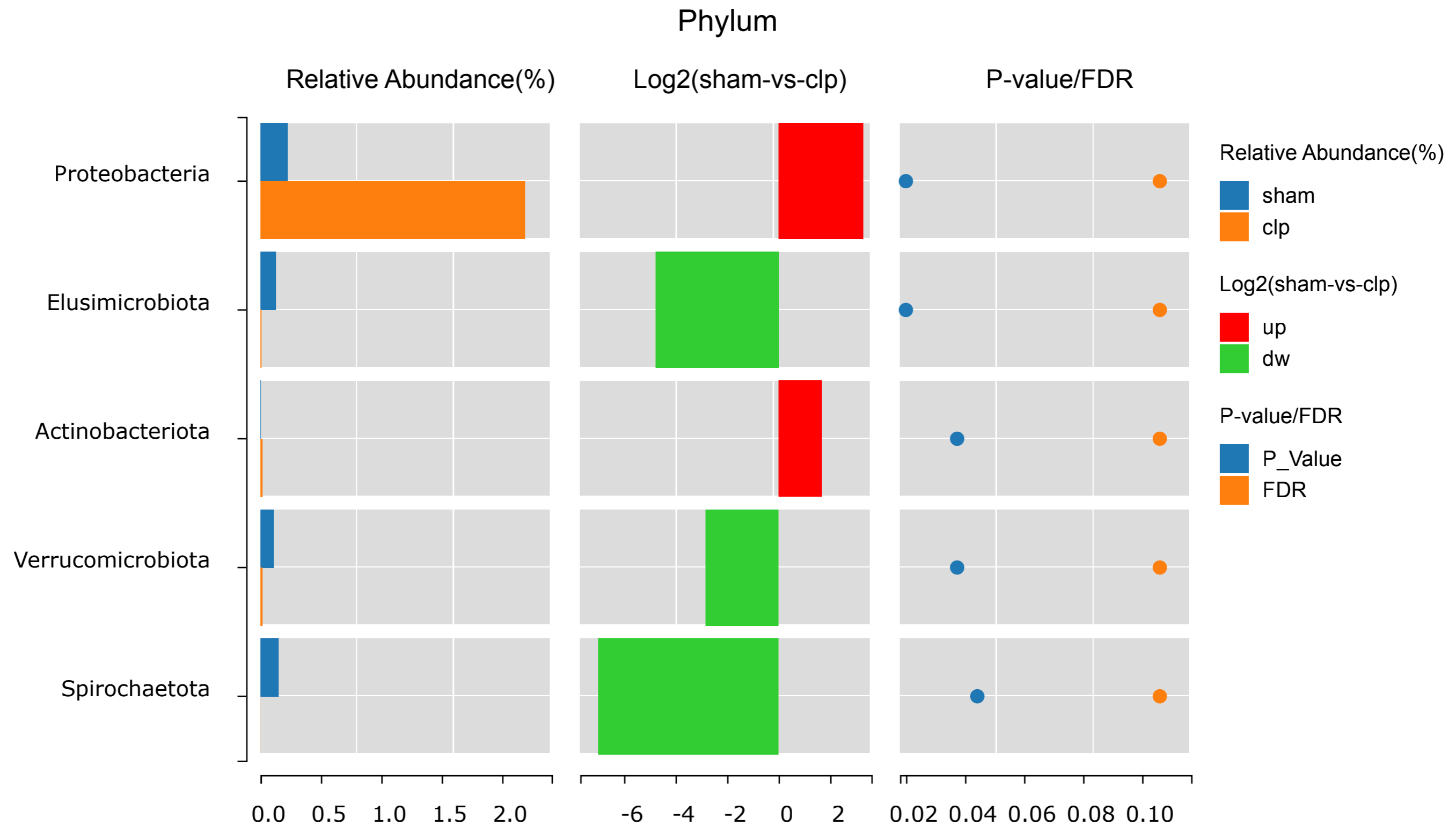

Supplement: Supplemental Information 4 [file peerj-11-15122-s004.zip › Samples without C1/Phylum.pdf]

P=0.19

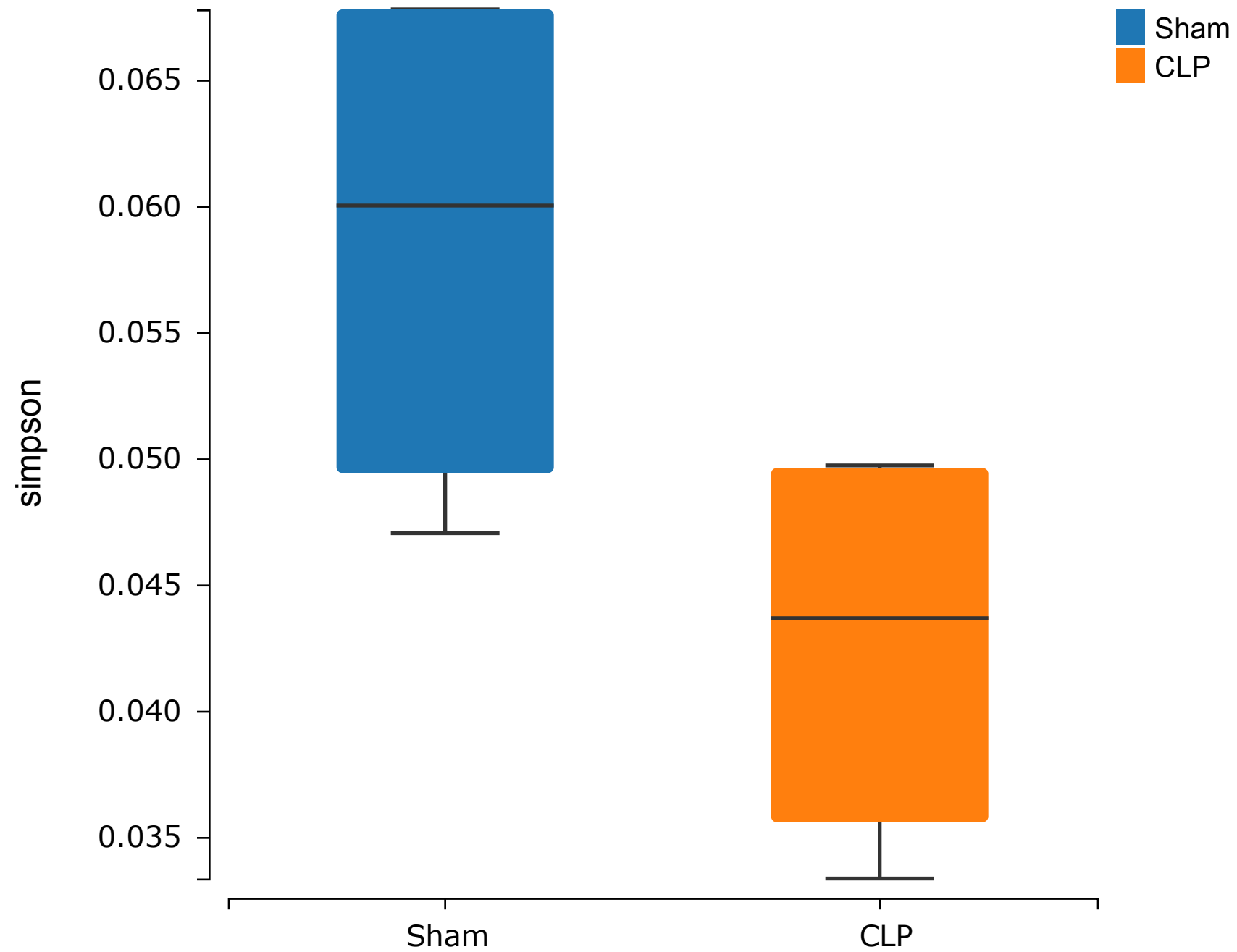

Supplement: Supplemental Information 4 [file peerj-11-15122-s004.zip › Samples without C1/Simpson.pdf]

P=0.016

Chao1

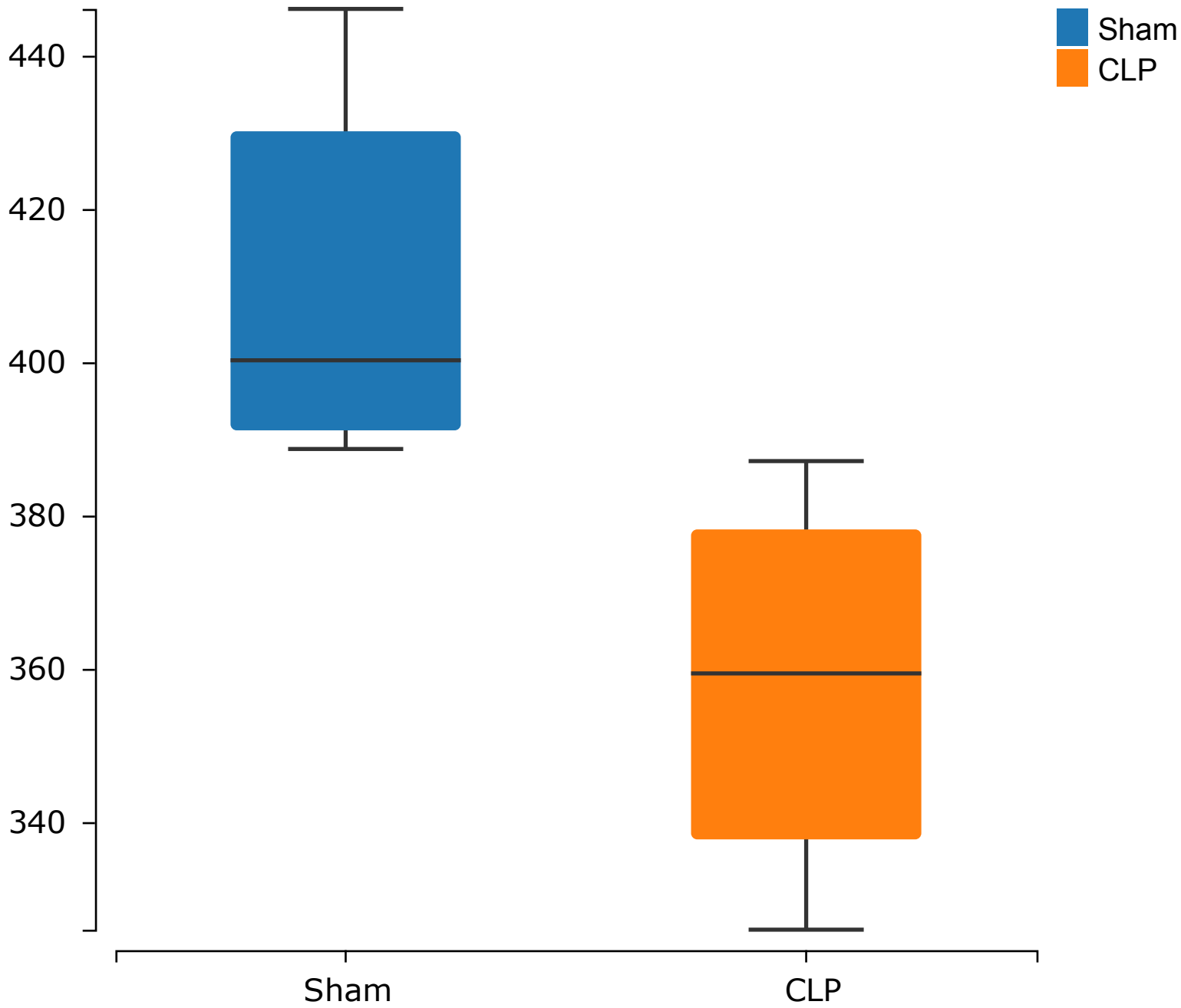

Supplement: Supplemental Information 4 [file peerj-11-15122-s004.zip › Samples without C1/Chao1.pdf]

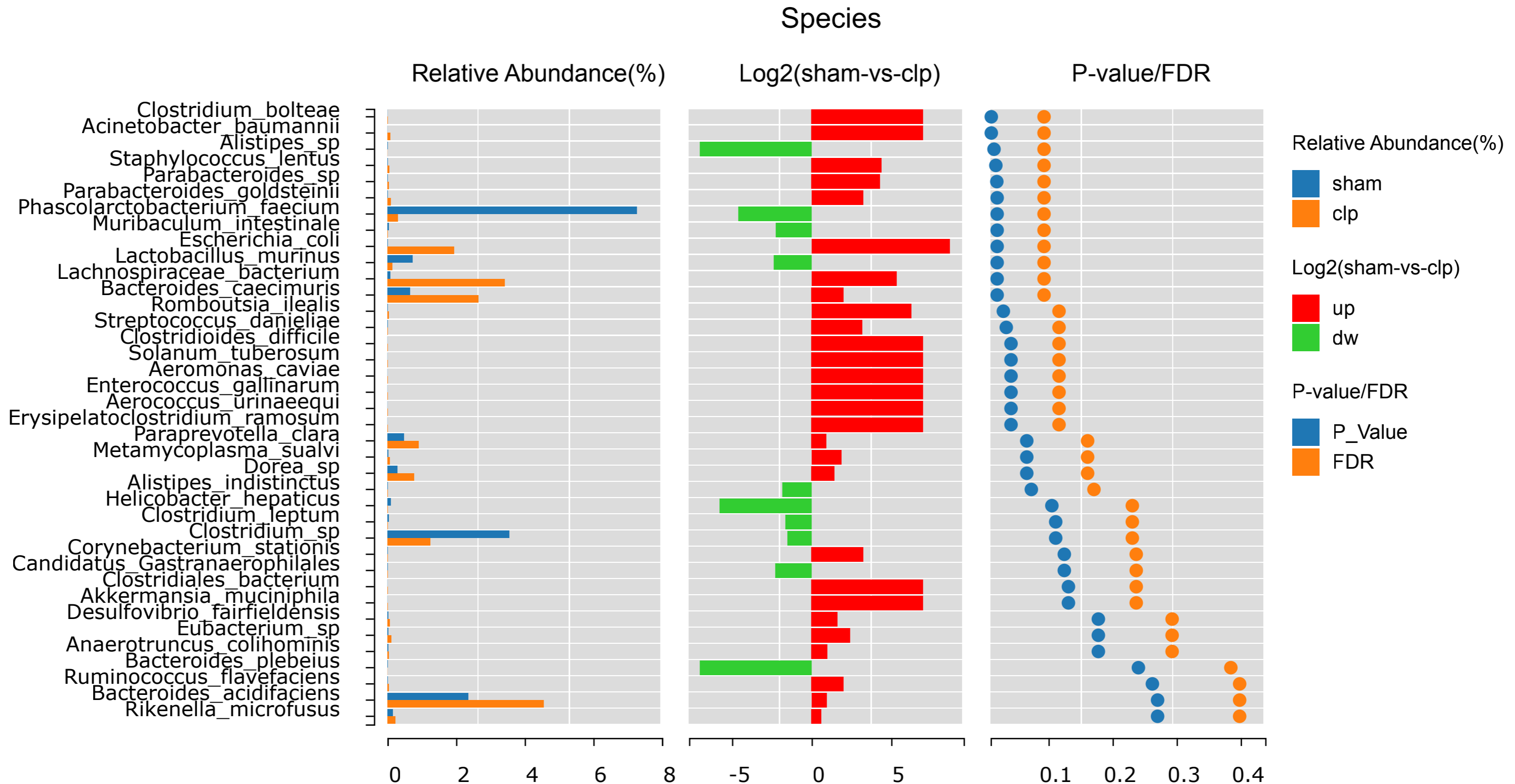

Supplement: Supplemental Information 4 [file peerj-11-15122-s004.zip › Samples without C1/Species.pdf]

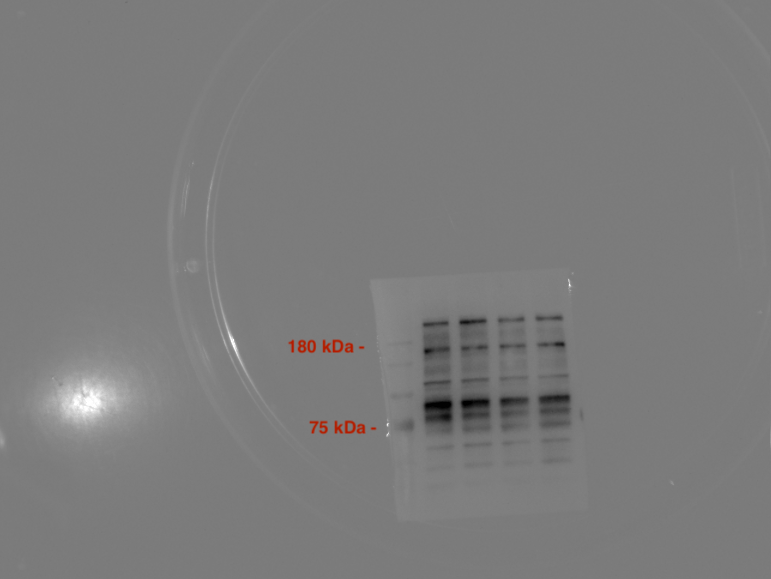

Supplement: Supplemental Information 5 — We marked the predicted band size of target proteins and some visible molecular weight markers on the images included in the figures. Other replicates are available at Figshare. [file peerj-11-15122-s005.zip › Gel Blots(in Fig)/gut/ZO1.tif]

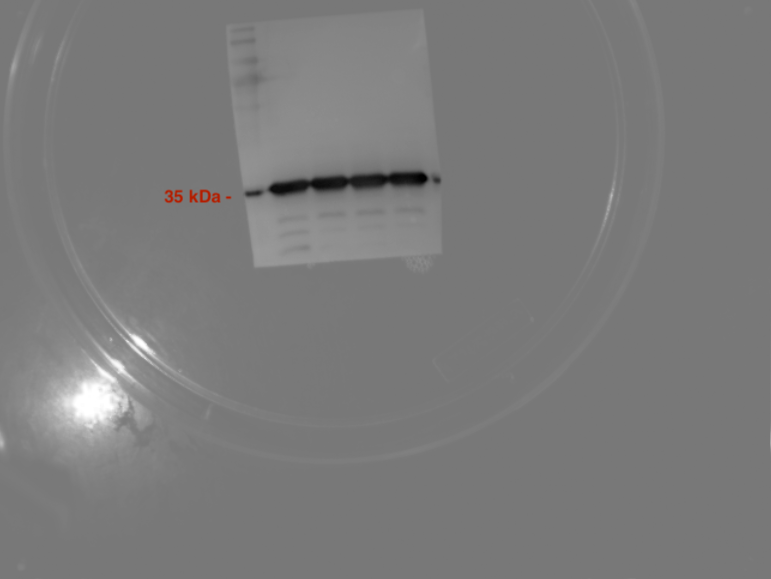

Supplement: Supplemental Information 5 — We marked the predicted band size of target proteins and some visible molecular weight markers on the images included in the figures. Other replicates are available at Figshare. [file peerj-11-15122-s005.zip › Gel Blots(in Fig)/gut/GAPDH.tif]

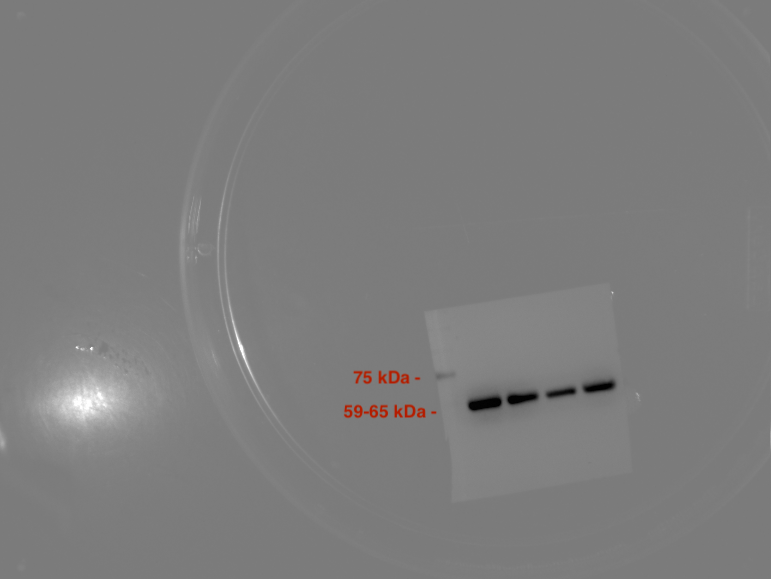

Supplement: Supplemental Information 5 — We marked the predicted band size of target proteins and some visible molecular weight markers on the images included in the figures. Other replicates are available at Figshare. [file peerj-11-15122-s005.zip › Gel Blots(in Fig)/gut/occludin.tif]

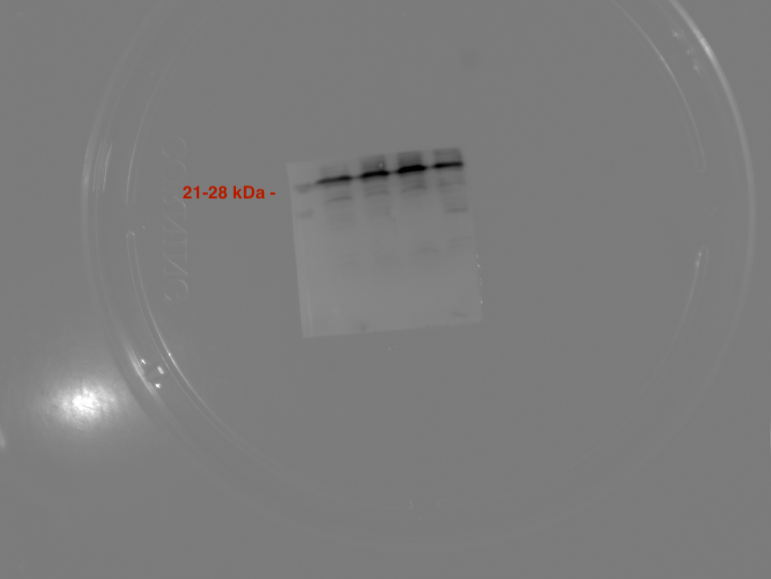

Supplement: Supplemental Information 5 — We marked the predicted band size of target proteins and some visible molecular weight markers on the images included in the figures. Other replicates are available at Figshare. [file peerj-11-15122-s005.zip › Gel Blots(in Fig)/Brain/Inflammatary factors/IL-6.tif]

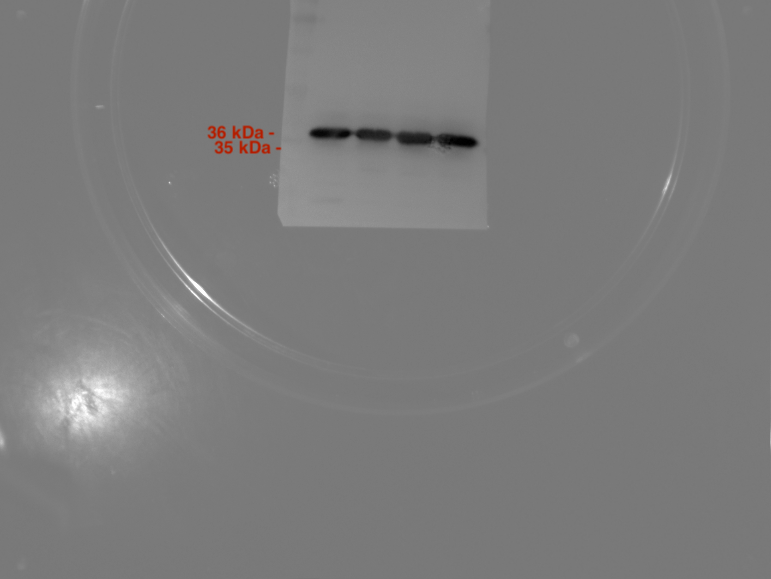

Supplement: Supplemental Information 5 — We marked the predicted band size of target proteins and some visible molecular weight markers on the images included in the figures. Other replicates are available at Figshare. [file peerj-11-15122-s005.zip › Gel Blots(in Fig)/Brain/Inflammatary factors/GAPDH.tif]

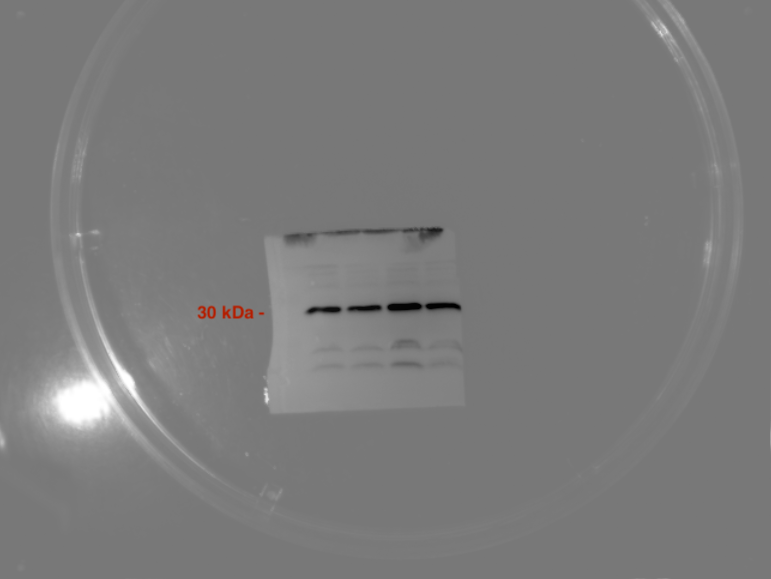

Supplement: Supplemental Information 5 — We marked the predicted band size of target proteins and some visible molecular weight markers on the images included in the figures. Other replicates are available at Figshare. [file peerj-11-15122-s005.zip › Gel Blots(in Fig)/Brain/Inflammatary factors/IL-1B.tif]

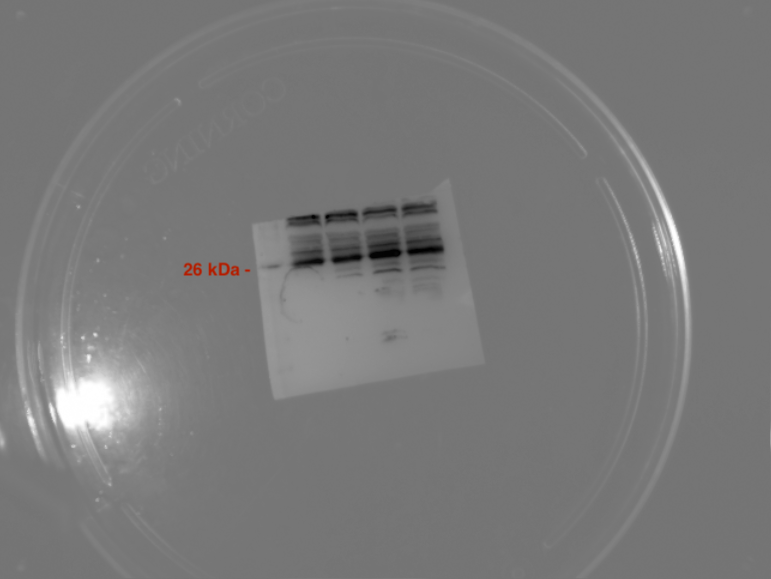

Supplement: Supplemental Information 5 — We marked the predicted band size of target proteins and some visible molecular weight markers on the images included in the figures. Other replicates are available at Figshare. [file peerj-11-15122-s005.zip › Gel Blots(in Fig)/Brain/Inflammatary factors/TNF-a.tif]

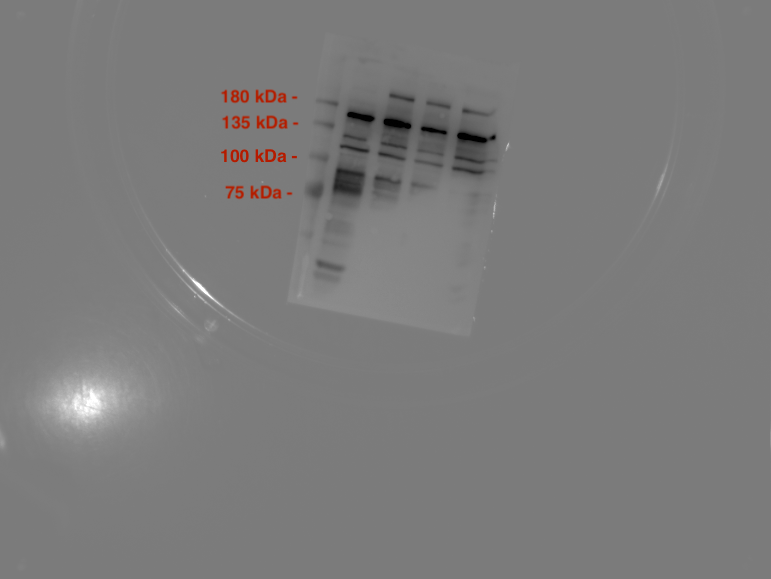

Supplement: Supplemental Information 5 — We marked the predicted band size of target proteins and some visible molecular weight markers on the images included in the figures. Other replicates are available at Figshare. [file peerj-11-15122-s005.zip › Gel Blots(in Fig)/Brain/TF/zo-1.tif]

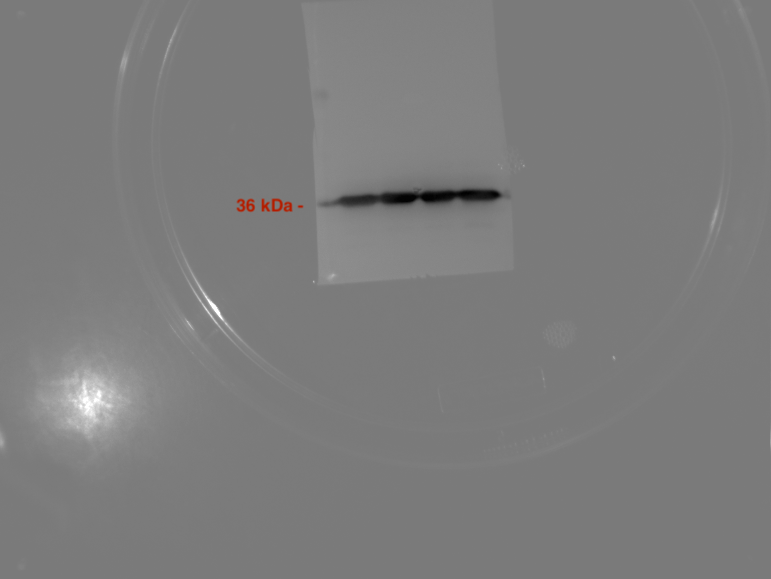

Supplement: Supplemental Information 5 — We marked the predicted band size of target proteins and some visible molecular weight markers on the images included in the figures. Other replicates are available at Figshare. [file peerj-11-15122-s005.zip › Gel Blots(in Fig)/Brain/TF/GAPDH.tif]

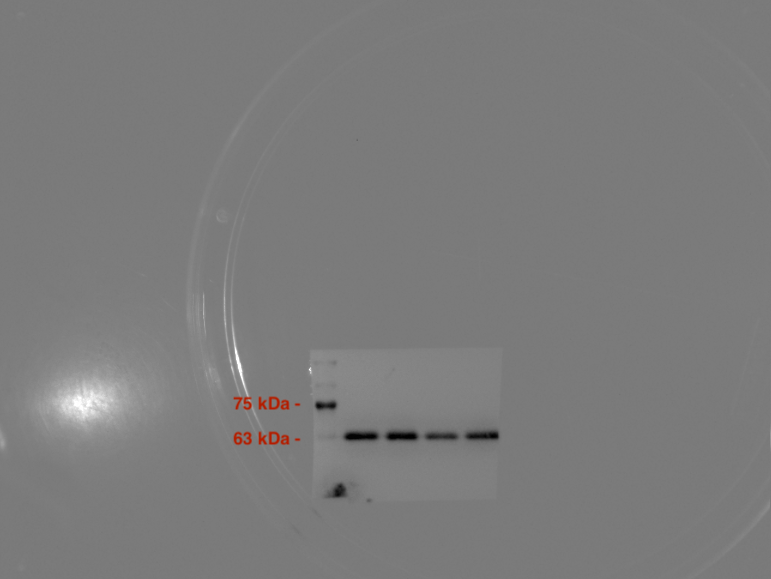

Supplement: Supplemental Information 5 — We marked the predicted band size of target proteins and some visible molecular weight markers on the images included in the figures. Other replicates are available at Figshare. [file peerj-11-15122-s005.zip › Gel Blots(in Fig)/Brain/TF/occludin.tif]
