# Supplementary material for: The modulatory effects of gut microbes and metabolites on blood–brain barrier integrity and brain function in sepsis-associated encephalopathy
Source: PeerJ. 2023 Mar 28;11:e15122. doi: 10.7717/peerj.15122 (PMC10064995; doi:10.7717/peerj.15122)
Supplement: Supplemental Information 4 [file peerj-11-15122-s004.zip › Samples without C1/Lefse_sham-clp.result.cladogram.pdf]

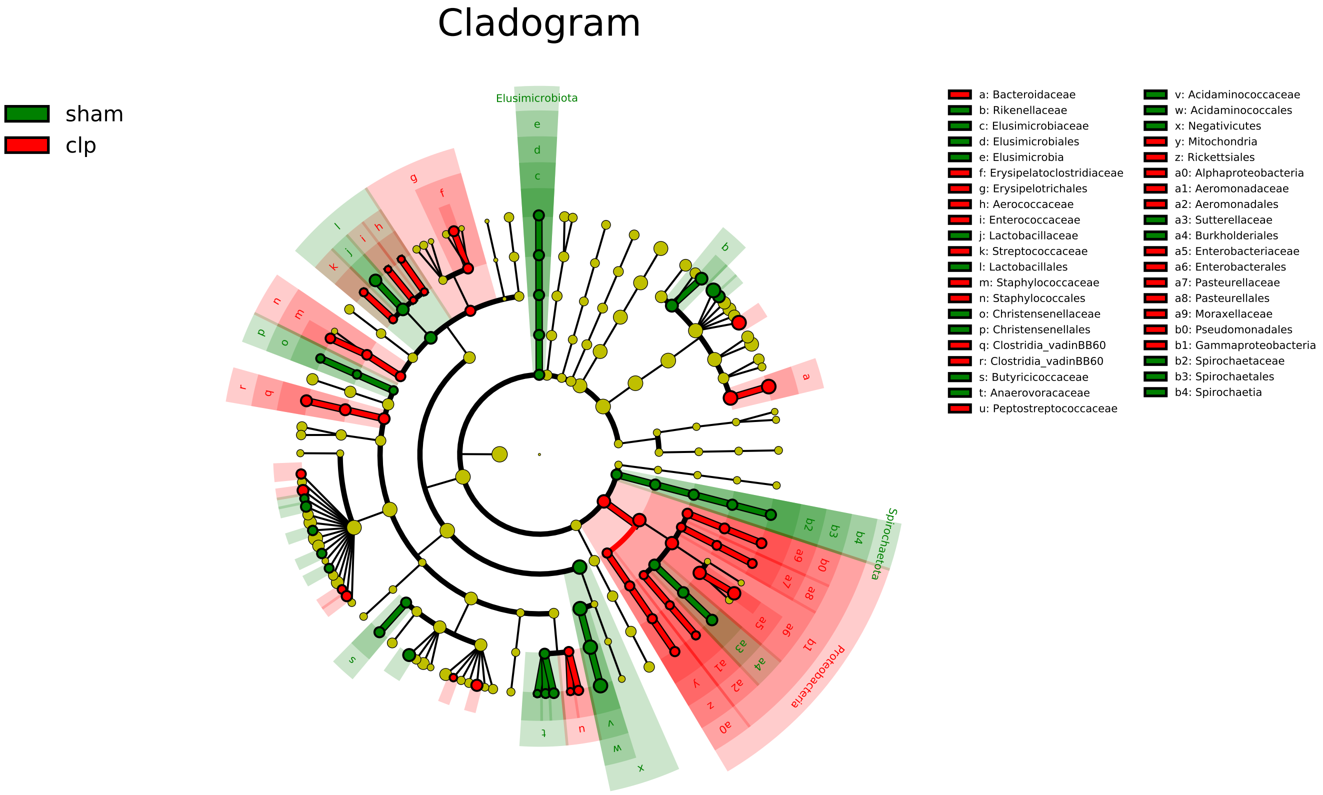
Cladogram
sham
clp
Elusimicrobiota
Spirochaetota
Proteobacteria
a: Bacteroidaceae
b: Rikenellaceae
c: Elusimicrobiaceae
d: Elusimicrobiales
e: Elusimicrobia
f: Erysipelatoclostridiaceae
g: Erysipelotrichales
h: Aerococcaceae
i: Enterococcaceae
j: Lactobacillaceae
k: Streptococcaceae
l: Lactobacillales
m: Staphylococcaceae
n: Staphylococcales
o: Christensenellaceae
p: Christensenellales
q: Clostridia_vadinBB60
r: Clostridia_vadinBB60
s: Butyricicoccaceae
t: Anaerovoracaceae
u: Peptostreptococcaceae
v: Acidaminococcaceae
w: Acidaminococcales
x: Negativicutes
y: Mitochondria
z: Rickettsiales
a0: Alphaproteobacteria
a1: Aeromonadaceae
a2: Aeromonadales
a3: Sutterellaceae
a4: Burkholderiales
a5: Enterobacteriaceae
a6: Enterobacterales
a7: Pasteurellaceae
a8: Pasteurellales
a9: Moraxellaceae
b0: Pseudomonadales
b1: Gammaproteobacteria
b2: Spirochaetaceae
b3: Spirochaetales
b4: Spirochaetia
