# Supplementary material for: The modulatory effects of gut microbes and metabolites on blood–brain barrier integrity and brain function in sepsis-associated encephalopathy
Source: PeerJ. 2023 Mar 28;11:e15122. doi: 10.7717/peerj.15122 (PMC10064995; doi:10.7717/peerj.15122)
Supplement: Supplemental Information 4 [file peerj-11-15122-s004.zip › Samples without C1/Genus.pdf]

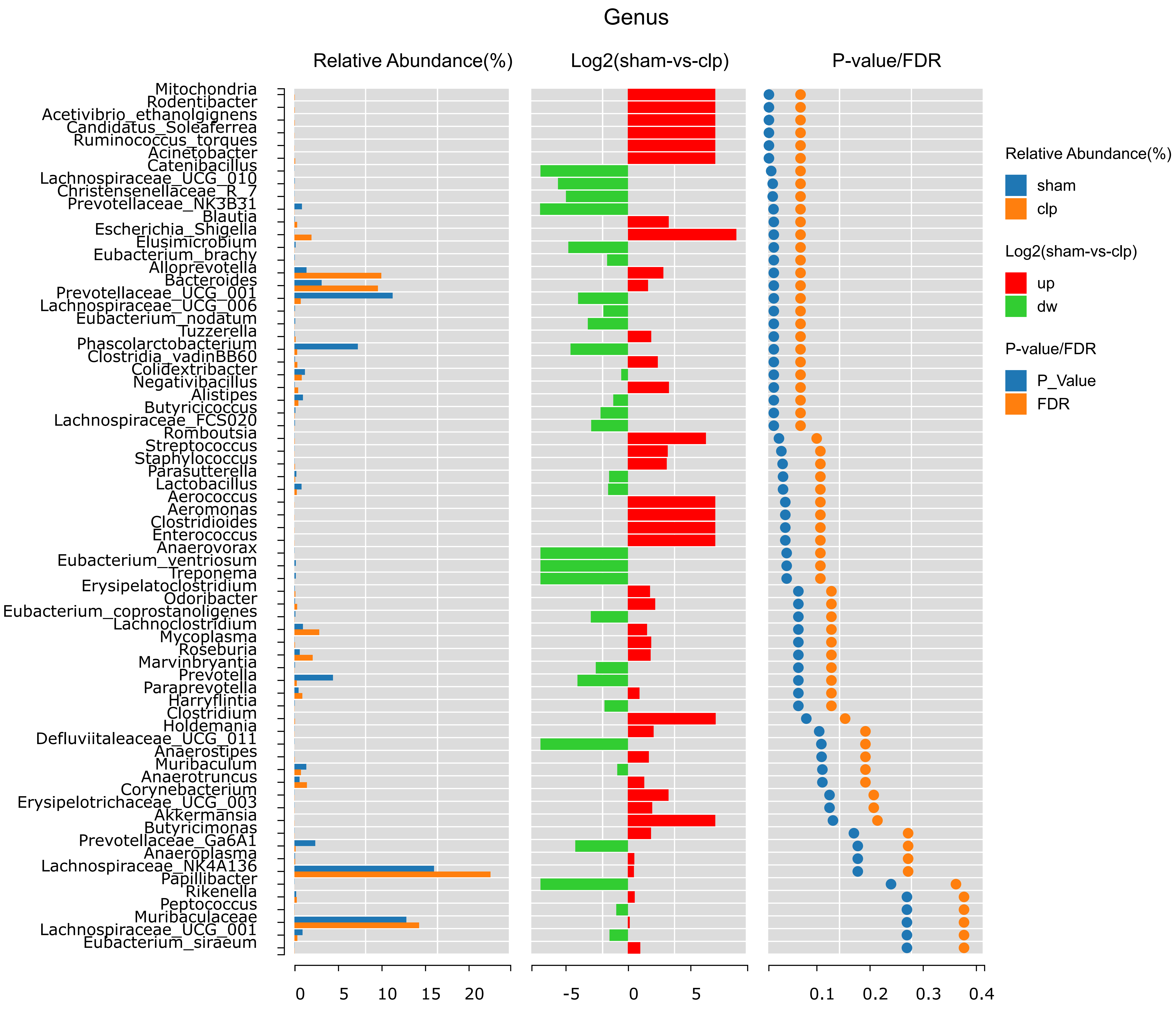
Genus
Relative Abundance(%)
Log2(sham-vs-clp)
P-value/FDR
Mitochondria
Rodentibacter
Acetivibrio_ethanolgignens
Candidatus_Soleaferrea
Ruminococcus_torques
Acinetobacter
Catenibacillus
Lachnospiraceae_UCG_010
Christensenellaceae_R_7
Prevotellaceae_NK3B31
Blautia
Escherichia_Shigella
Elusimicrobium
Eubacterium_brachy
Alloprevotella
Bacteroides
Prevotellaceae_UCG_001
Lachnospiraceae_UCG_006
Eubacterium_nodatum
Tuzzerella
Phascolarctobacterium
Clostridia_vadinBB60
Colidextribacter
Negativibacillus
Alistipes
Butyricicoccus
Lachnospiraceae_FCS020
Romboutsia
Streptococcus
Staphylococcus
Parasutterella
Lactobacillus
Aerococcus
Aeromonas
Clostridioides
Enterococcus
Anaerovorax
Eubacterium_ventriosum
Treponema
Erysipelatoclostridium
Odoribacter
Eubacterium_coprostanoligenes
Lachnoclostridium
Mycoplasma
Roseburia
Marvinbryantia
Prevotella
Paraprevotella
Harryflintia
Clostridium
Holdemania
Defluviitaleaceae_UCG_011
Anaerostipes
Muribaculum
Anaerotruncus
Corynebacterium
Erysipelotrichaceae_UCG_003
Akkermansia
Butyricimonas
Prevotellaceae_Ga6A1
Anaeroplasma
Lachnospiraceae_NK4A136
Papillibacter
Rikenella
Peptococcus
Muribaculaceae
Lachnospiraceae_UCG_001
Eubacterium_siraeum
0
5
10
15
20
-5
0
5
0.1
0.2
0.3
0.4
Relative Abundance(%)
sham
clp
Log2(sham-vs-clp)
up
dw
P-value/FDR
P_Value
FDR
